# Supplementary material for: A Likelihood Approach for Real-Time Calibration of Stochastic Compartmental Epidemic Models
Source: PLoS Comput Biol. 2017 Jan 17;13(1):e1005257. doi: 10.1371/journal.pcbi.1005257 (PMC5240920; doi:10.1371/journal.pcbi.1005257)
Supplement: S1 File — (TAR.GZ) [file pcbi.1005257.s014.tar.gz › HSPH_Online-SI-Revision/PF08/n100-mild/n100-mild_PF_table-4.pdf]

1	R0	{1.24125, 3.38982}	{2.17605, 19.4377}	{{1.83826, 16.9976}, {2.28986, 20.}}	{75.311, 473.414}	inf	1007.	5929.9	{4697.99, 6760.49}	488.867	0.766499	newly	2409.	2506.26	{2236., 2863.}	8.05812	0.033849	Reff	1.16056	1.91878	{1.72201, 2.05194}	65.333	ar	34926.	92798.4	{84402., 95569.}	165.7
2	R0	{1.29554, 4.51878}	{2.14289, 19.6965}	{{2.10928, 20.}, {2.22655, 20.}}	{65.4051, 335.881}	inf	1718.	7921.62	{7001.34, 8794.28}	361.096	0.660537	newly	2989.	3225.65	{2775., 3575.}	10.5564	0.0434971	Reff	1.16829	1.79045	{1.76408, 1.84905}	53.2537	ar	42828.	90118.1	{89600., 91773.}	110.419
3	R0	{1.2443, 3.64292}	{1.91739, 16.1589}	{{1.69372, 12.1985}, {2.18933, 20.}}	{54.0936, 343.57}	inf	1255.	4916.04	{3778.83, 6128.44}	291.717	0.58505	newly	2853.	2507.11	{2211., 2796.}	12.3235	0.0589333	Reff	1.15569	1.73245	{1.56762, 1.98357}	49.906	ar	37087.	83416.6	{75458., 91095.}	124.921
4	R0	{1.38465, 3.43331}	{2.87671, 17.6095}	{{2.35557, 14.3612}, {3., 20.}}	{107.757, 412.901}	inf	1365.	6700.41	{5422.34, 7341.39}	390.873	0.688042	newly	3916.	3546.83	{3331., 3754.}	9.49566	0.0438451	Reff	1.30897	2.45129	{2.18376, 2.70582}	87.2696	ar	49511.	97353.2	{92444., 99224.}	96.6294
5	R0	{1.25409, 4.1068}	{1.85014, 13.4377}	{{1.73503, 11.801}, {2.17645, 18.4258}}	{47.5281, 227.206}	inf	1456.	5107.58	{4394.23, 6924.45}	250.796	0.538844	newly	2839.	3168.35	{2842., 3486.}	12.168	0.048999	Reff	1.15606	1.67156	{1.57174, 1.90318}	44.5915	ar	38571.	80985.9	{77365., 90121.}	109.966
6	R0	{1.28821, 4.2016}	{2.18085, 19.6778}	{{2.0566, 20.}, {2.28543, 20.}}	{69.2929, 368.34}	inf	1742.	8108.95	{7187.23, 8790.56}	365.497	0.665867	newly	3382.	3351.54	{2965., 3615.}	5.49793	0.0243596	Reff	1.16363	1.86365	{1.75489, 1.93505}	60.1591	ar	41468.	90362.9	{88245., 92190.}	117.91
7	R0	{1.24369, 2.56007}	{2.67117, 19.5885}	{{2.42918, 19.5975}, {2.80478, 20.}}	{114.778, 665.154}	inf	601.	4237.81	{3533.15, 4743.31}	605.127	0.843523	newly	2262.	1985.85	{1714., 2219.}	12.7714	0.0613819	Reff	1.20376	2.50473	{2.2887, 2.62592}	108.077	ar	38603.	94059.3	{91628., 96047.}	143.658
8	R0	{1.232, 3.53942}	{2.22081, 19.7728}	{{1.94472, 18.3}, {2.27895, 20.}}	{80.2603, 458.646}	inf	1387.	8445.83	{6815.74, 9347.49}	508.928	0.78213	newly	3108.	3440.72	{3069., 3782.}	12.2181	0.0496325	Reff	1.11866	1.82361	{1.7256, 1.85263}	63.0179	ar	34555.	93814.7	{88004., 95358.}	171.494
9	R0	{1.36262, 3.85631}	{2.77381, 19.6999}	{{2.59234, 20.}, {2.87741, 20.}}	{103.564, 410.849}	inf	1592.	8196.58	{7630.87, 8829.63}	414.861	0.709509	newly	3831.	3733.35	{3437., 4023.}	4.91073	0.0218666	Reff	1.27209	2.4311	{2.28071, 2.50597}	91.1113	ar	48410.	96181.3	{94283., 97713.}	98.6806
10	R0	{1.28702, 4.67968}	{1.79073, 16.6506}	{{1.71592, 15.1263}, {1.99334, 20.}}	{39.1374, 255.807}	inf	1874.	6609.27	{6030.18, 8169.73}	252.683	0.545189	newly	3114.	3097.76	{2852., 3261.}	4.22864	0.0186287	Reff	1.13924	1.52889	{1.46907, 1.63802}	34.2019	ar	41092.	78695.7	{75939., 85448.}	91.5109
11	R0	{1.35752, 3.47129}	{2.06634, 17.0781}	{{2.02624, 16.4324}, {2.14365, 19.476}}	{52.2146, 391.982}	inf	974.	4031.56	{3710.65, 4539.51}	313.918	0.616057	newly	2552.	2008.47	{1820., 2215.}	21.2982	0.105191	Reff	1.29461	1.93091	{1.90189, 2.00228}	49.1493	ar	44669.	86027.6	{84678., 88249.}	92.5891
12	R0	{1.35929, 5.83647}	{2.04954, 18.9626}	{{1.78444, 16.6437}, {2.20042, 20.}}	{50.7803, 224.899}	inf	2459.	8135.8	{6466.95, 9388.16}	230.858	0.515042	newly	3330.	3370.46	{2994., 3744.}	6.55916	0.0281943	Reff	1.20052	1.66862	{1.54806, 1.74132}	38.991	ar	46715.	87735.4	{80677., 91626.}	87.81
13	R0	{1.28019, 3.29908}	{2.04262, 14.1805}	{{1.79532, 11.4471}, {2.36849, 20.}}	{59.556, 329.832}	inf	1185.	4574.53	{3578.76, 6718.32}	286.036	0.571191	newly	3104.	2793.39	{2559., 2954.}	12.5815	0.0577775	Reff	1.21037	1.89034	{1.68518, 2.15871}	56.1789	ar	40941.	84765.6	{78726., 93355.}	107.043
14	R0	{1.36661, 3.48318}	{2.99436, 19.9248}	{{3., 20.}, {3., 20.}}	{119.108, 472.028}	inf	1869.	10668.7	{10313.9, 11020.7}	470.823	0.756076	newly	4569.	4869.74	{4691., 5017.}	6.93543	0.0290372	Reff	1.26507	2.54148	{2.51596, 2.58241}	100.896	ar	48672.	99391.9	{98806., 100020.}	104.208
15	R0	{1.35865, 3.84123}	{2.19424, 17.2975}	{{2.17848, 17.1059}, {2.20672, 17.4941}}	{61.5016, 350.31}	inf	1321.	5134.67	{4988.9, 5224.99}	288.696	0.589492	newly	3065.	2545.43	{2397., 2658.}	16.9517	0.0810023	Reff	1.28034	2.0161	{2.00345, 2.02535}	57.4664	ar	48598.	88906.6	{88561., 89279.}	82.9429
16	R0	{1.22879, 3.98329}	{1.81007, 17.8973}	{{1.71267, 16.7617}, {1.99476, 20.}}	{47.3054, 349.311}	inf	1186.	5488.92	{4890.08, 6242.65}	362.809	0.663359	newly	2363.	2420.42	{2213., 2581.}	5.17562	0.0220856	Reff	1.12545	1.62843	{1.53953, 1.78923}	44.692	ar	34405.	81565.	{77550., 88248.}	137.073
17	R0	{1.34925, 3.64524}	{2.77116, 19.901}	{{2.52482, 20.}, {2.91599, 20.}}	{105.385, 445.945}	inf	1082.	6277.08	{5647.75, 6817.48}	480.137	0.761204	newly	2774.	2888.1	{2505., 3122.}	8.08868	0.0344689	Reff	1.28476	2.5226	{2.32077, 2.65051}	96.3483	ar	46871.	96208.7	{93626., 98042.}	105.263
18	R0	{1.33054, 2.25275}	{2.89283, 17.1429}	{{2.59592, 14.4796}, {3., 20.}}	{117.417, 660.977}	inf	387.	2486.15	{1977.81, 2932.77}	542.416	0.800839	newly	1979.	1512.99	{1147., 1928.}	23.8267	0.12551	Reff	1.30961	2.78254	{2.49496, 2.91901}	112.471	ar	44336.	95426.6	{93166., 97156.}	115.235
19	R0	{1.28394, 3.69859}	{2.1083, 19.7305}	{{1.81028, 17.689}, {2.22716, 20.}}	{64.2057, 433.461}	inf	1262.	6127.68	{5057.61, 6954.64}	385.553	0.68263	newly	2837.	2497.41	{2063., 2756.}	12.0278	0.0584114	Reff	1.18844	1.79695	{1.63143, 1.83034}	51.2023	ar	39558.	88074.8	{79963., 90411.}	122.647
20	R0	{1.2883, 2.26493}	{2.59783, 15.6074}	{{1.88159, 11.4933}, {3., 20.}}	{101.648, 589.09}	inf	248.	1605.	{985.429, 2248.24}	547.177	0.789427	newly	1268.	1033.87	{638., 1554.}	28.6491	0.149898	Reff	1.27318	2.504	{1.8374, 2.94017}	96.6735	ar	40768.	90970.6	{78964., 96894.}	123.142
21	R0	{1.34098, 2.78621}	{2.61991, 15.2835}	{{2.10294, 10.5809}, {3., 20.}}	{95.3724, 448.539}	inf	675.	3580.21	{2488.75, 4822.76}	430.402	0.699211	newly	2390.	2317.19	{1819., 2727.}	11.2013	0.0513281	Reff	1.30397	2.45746	{2.02347, 2.80571}	88.4598	ar	44605.	90913.7	{84655., 96903.}	103.819
22	R0	{1.33783, 5.95911}	{1.79313, 16.7462}	{{1.68066, 14.8804}, {1.85887, 17.8248}}	{34.033, 181.019}	inf	2154.	6629.93	{5699.49, 7321.39}	207.796	0.485628	newly	2912.	3098.87	{2877., 3297.}	7.29979	0.0303661	Reff	1.18802	1.55887	{1.48061, 1.6145}	31.2155	ar	45418.	82102.8	{77715., 84918.}	80.7716
23	R0	{1.33359, 3.3069}	{2.12417, 16.7496}	{{1.77421, 11.804}, {2.21488, 18.0126}}	{59.2821, 406.506}	inf	965.	3940.59	{2972.28, 4252.12}	308.351	0.608152	newly	2751.	2034.35	{1896., 2177.}	26.0505	0.131823	Reff	1.27359	2.00004	{1.68001, 2.08165}	57.0392	ar	46245.	87648.1	{77466., 89987.}	89.5299
24	R0	{1.30588, 2.56123}	{2.66217, 18.479}	{{2.33948, 16.4616}, {3., 20.}}	{103.86, 621.488}	inf	683.	4268.41	{3428.57, 5523.21}	524.951	0.786297	newly	2554.	2151.13	{1827., 2586.}	17.0004	0.0827892	Reff	1.26499	2.48481	{2.22537, 2.77545}	96.4287	ar	42383.	95260.6	{91119., 98325.}	124.761
25	R0	{1.29813, 4.51849}	{1.91616, 19.9978}	{{1.88165, 20.}, {1.9482, 20.}}	{47.6092, 342.576}	inf	1351.	5403.18	{5016.86, 5724.54}	299.939	0.601404	newly	2397.	2137.52	{1944., 2290.}	10.8252	0.0505194	Reff	1.19921	1.7093	{1.68475, 1.73289}	42.5354	ar	43371.	82362.3	{81227., 83219.}	89.9017
26	R0	{1.33231, 3.99953}	{2.24088, 20.}	{{2.1649, 20.}, {2.30931, 20.}}	{68.1952, 400.059}	inf	1549.	7040.29	{6626.86, 7435.4}	354.505	0.657139	newly	3228.	2904.01	{2717., 3095.}	10.11	0.0467955	Reff	1.2303	1.95276	{1.90661, 1.9949}	58.7225	ar	44733.	93684.3	{92216., 94952.}	109.43
27	R0	{1.30555, 4.61445}	{1.933, 20.}	{{1.89996, 20.}, {1.95711, 20.}}	{48.0601, 333.421}	inf	1634.	5669.17	{5276.21, 5976.3}	246.951	0.53972	newly	2878.	2250.7	{2066., 2381.}	21.7964	0.107423	Reff	1.20568	1.72328	{1.70323, 1.74075}	42.9303	ar	42624.	83594.5	{82493., 84588.}	96.1207
28	R0	{1.26819, 5.34969}	{2.00429, 18.5882}	{{1.74843, 15.9243}, {2.20315, 20.}}	{58.0431, 247.463}	inf	1779.	6209.67	{4730.25, 7410.94}	249.054	0.536464	newly	2612.	2661.12	{2327., 2952.}	8.53292	0.0366406	Reff	1.1566	1.72723	{1.5737, 1.83499}	49.3363	ar	38946.	87060.4	{79384., 92513.}	123.541
29	R0	{1.19925, 3.00394}	{2.1577, 19.5495}	{{2.00074, 18.0158}, {2.28039, 20.}}	{79.9208, 550.794}	inf	770.	5430.45	{4864.64, 6108.53}	605.253	0.845259	newly	2181.	2296.06	{2025., 2546.}	9.12976	0.0382612	Reff	1.12966	1.947	{1.81475, 2.02168}	72.3534	ar	31727.	89911.2	{86451., 92170.}	183.39
30	R0	{1.35749, 3.75483}	{2.66912, 18.9531}	{{1.82932, 12.0098}, {2.85215, 20.}}	{96.6216, 404.765}	inf	1440.	7274.11	{3699.08, 8264.26}	405.146	0.689798	newly	3590.	3423.51	{2532., 3756.}	7.17521	0.0355071	Reff	1.28281	2.39831	{1.72088, 2.54274}	86.9567	ar	48244.	95129.4	{80668., 97932.}	97.1838
31	R0	{1.27131, 4.2161}	{2.20827, 19.9733}	{{2.12838, 20.}, {2.29484, 20.}}	{73.7001, 373.738}	inf	1285.	6237.67	{5744.64, 6606.51}	385.422	0.685449	newly	2456.	2574.43	{2365., 2744.}	6.31637	0.026454	Reff	1.18819	1.95046	{1.91097, 1.99047}	64.154	ar	38299.	93103.6	{91689., 94848.}	143.097
32	R0	{1.31827, 6.18119}	{1.93949, 18.183}	{{1.75891, 16.4074}, {2.17953, 20.}}	{47.1241, 194.167}	inf	2473.	7526.36	{6316.42, 9298.96}	204.341	0.478821	newly	3092.	3240.86	{2898., 3680.}	7.57245	0.0313226	Reff	1.16063	1.61649	{1.52858, 1.72737}	39.277	ar	45262.	84809.	{79552., 90917.}	87.3734
33	R0	{1.20398, 1.35167}	{2.21498, 16.7123}	{{1.50724, 9.59729}, {3., 20.}}	{83.9716, 1136.42}	inf	104.	944.944	{397.955, 1590.19}	808.6	0.909884	newly	1068.	511.69	{255., 792.}	52.4036	0.364154	Reff	1.19578	2.17012	{1.48518, 2.91569}	81.4812	ar	33329.	82010.	{61631., 95964.}	146.062
34	R0	{1.32776, 3.146}	{2.75706, 20.}	{{2.69417, 20.}, {2.80077, 20.}}	{107.648, 535.728}	inf	1228.	7502.43	{6763.45, 7973.61}	510.947	0.785259	newly	3687.	3363.03	{3013., 3600.}	8.89802	0.041302	Reff	1.25318	2.43077	{2.38557, 2.46574}	93.9681	ar	44872.	94997.1	{94121., 95712.}	111.707
35	R0	{1.279, 1.11336}	{2.00804, 16.9213}	{{1.44739, 12.5395}, {2.63121, 20.}}	{57.0007, 1419.84}	inf	39.	545.833	{252.356, 862.124}	1299.57	1.09928	newly	625.	275.54	{141., 409.}	56.0128	0.394664	Reff	1.27363	1.98155	{1.44397, 2.60314}	55.5832	ar	41033.	77101.1	{57644., 92837.}	87.9003
36	R0	{1.20029, 1.94717}	{2.84813, 17.6203}	{{2.5427, 14.6706}, {3., 20.}}	{137.287, 804.918}	inf	272.	2463.03	{1986.04, 2871.33}	805.527	0.948445	newly	1470.	1437.58	{1041., 1843.}	16.985	0.0767373	Reff	1.18231	2.7511	{2.42924, 2.92338}	132.689	ar	33234.	95254.2	{92658., 97472.}	186.617
37	R0	{1.33216, 3.69685}	{2.10002, 17.6177}	{{2.03354, 17.0766}, {2.13536, 17.9613}}	{57.6405, 376.559}	inf	1487.	6226.25	{5751.69, 6475.33}	318.712	0.621552	newly	3432.	2936.92	{2697., 3120.}	14.4254	0.0682928	Reff	1.23359	1.8739	{1.83284, 1.90534}	51.9056	ar	45235.	87260.1	{85890., 88044.}	92.904
38	R0	{1.25434, 2.04314}	{2.16494, 15.728}	{{1.56054, 10.8605}, {2.87633, 20.}}	{72.5963, 669.795}	inf	320.	1233.89	{660.484, 1772.81}	285.59	0.558786	newly	1505.	710.91	{406., 976.}	52.7635	0.347231	Reff	1.23694	2.08053	{1.51046, 2.78193}	68.1997	ar	38662.	83102.7	{65584., 95336.}	114.947
39	R0	{1.27466, 3.0741}	{2.47941, 16.2365}	{{2.16615, 12.8133}, {3., 20.}}	{94.5157, 428.17}	inf	1446.	7241.98	{5505.51, 9402.8}	400.829	0.692695	newly	3912.	3999.64	{3658., 4391.}	6.36094	0.0269857	Reff	1.18848	2.18338	{1.95051, 2.59155}	83.7122	ar	40181.	94918.5	{90179., 99652.}	136.227
40	R0	{1.33045, 2.68154}	{3., 16.6572}	{{3., 15.6176}, {3., 20.}}	{125.488, 521.18}	inf	888.	5303.59	{4898.4, 5641.27}	497.252	0.775582	newly	3300.	3322.98	{2686., 3658.}	8.2	0.0364479	Reff	1.28496	2.81656	{2.79811, 2.83597}	119.194	ar	45951.	98859.1	{98259., 99450.}	115.14
41	R0	{1.37003, 2.83984}	{2.74929, 17.5601}	{{2.26289, 14.8251}, {3., 20.}}	{100.674, 518.347}	inf	521.	2634.68	{2186.23, 2958.14}	405.697	0.695279	newly	2106.	1520.99	{1135., 2125.}	29.0622	0.158593	Reff	1.34277	2.64528	{2.10545, 2.89898}	97.0025	ar	48926.	93909.1	{88377., 97410.}	91.941
42	R0	{1.3003, 2.94885}	{2.84355, 19.8779}	{{2.5487, 20.}, {2.97507, 20.}}	{118.684, 574.091}	inf	940.	5426.5	{4927.87, 5897.26}	477.288	0.759975	newly	2995.	2556.47	{2290., 2769.}	14.6421	0.0700541	Reff	1.24816	2.63219	{2.37864, 2.74515}	110.886	ar	44237.	97051.5	{94173., 98399.}	119.39
43	R0	{1.33756, 2.92653}	{2.82977, 18.3993}	{{2.30977, 14.9593}, {3., 20.}}	{111.562, 528.707}	inf	626.	2615.28	{2134.29, 2932.07}	317.776	0.617044	newly	1978.	1430.92	{1069., 2037.}	30.2053	0.161891	Reff	1.31066	2.7274	{2.26035, 2.90709}	108.094	ar	47342.	95409.6	{89839., 97541.}	101.533
44	R0	{1.35801, 2.92276}	{2.85278, 18.2006}	{{2.3112, 14.755}, {3., 20.}}	{110.07, 522.719}	inf	645.	3290.79	{2456.04, 3667.08}	410.2	0.70252	newly	2455.	1746.51	{1500., 1963.}	28.8591	0.14998	Reff	1.32668	2.64599	{2.21823, 2.88505}	99.4442	ar	47940.	96003.6	{90523., 98422.}	100.258
45	R0	{1.31342, 4.02649}	{1.92878, 14.014}	{{1.69801, 11.1771}, {2.38295, 20.}}	{46.8516, 248.045}	inf	1370.	4774.7	{3630.65, 7084.64}	248.518	0.526105	newly	2863.	2861.53	{2543., 3221.}	7.01467	0.0304962	Reff	1.22662	1.75381	{1.56839, 2.09843}	42.9798	ar	42995.	81050.2	{74053., 92210.}	88.5108
46	R0	{1.37919, 3.75889}	{2.69643, 19.2798}	{{2.4899, 19.3258}, {2.82451, 20.}}	{95.5081, 412.913}	inf	1025.	4745.37	{4504.67, 5353.87}	362.963	0.657605	newly	2667.	2273.16	{2061., 2497.}	14.7844	0.0710787	Reff	1.32725	2.5257	{2.31598, 2.6406}	90.2954	ar	49157.	94867.5	{93721., 96963.}	92.9889
47	R0	{1.37458, 3.56117}	{2.74149, 19.9742}	{{2.65583, 20.}, {2.81704, 20.}}	{99.4421, 460.889}	inf	1179.	5936.69	{5555.22, 6379.43}	403.536	0.701507	newly	3193.	2704.18	{2538., 2876.}	15.3091	0.0726749	Reff	1.31164	2.49345	{2.40866, 2.56961}	90.102	ar	48478.	95001.7	{93894., 96227.}	95.9687
48	R0	{1.33393, 3.78296}	{1.96433, 20.}	{{1.93945, 20.}, {1.98532, 20.}}	{47.2591, 428.687}	inf	1368.	4917.92	{4685.95, 5117.87}	259.497	0.555513	newly	3101.	1981.7	{1874., 2078.}	36.0948	0.194785	Reff	1.2473	1.79118	{1.77227, 1.80392}	43.6039	ar	44885.	84559.5	{83717., 85208.}	88.3915
49	R0	{1.24918, 2.39046}	{2.25987, 17.3365}	{{1.8871, 13.0479}, {2.47154, 20.}}	{80.908, 625.235}	inf	662.	4200.26	{3016.65, 5172.53}	534.48	0.792183	newly	2523.	2119.44	{1894., 2355.}	16.0951	0.0779246	Reff	1.19869	2.10401	{1.79135, 2.29527}	75.526	ar	35969.	88623.9	{80806., 93271.}	146.39
50	R0	{1.28281, 2.3032}	{2.97579, 19.603}	{{2.9447, 17.8858}, {3., 20.}}	{131.974, 751.121}	inf	746.	5740.08	{5211.49, 6265.54}	669.448	0.884385	newly	2869.	2791.47	{2601., 2987.}	5.15267	0.0229317	Reff	1.23601	2.71832	{2.52125, 2.79696}	119.926	ar	40223.	97869.8	{97271., 98632.}	143.318
